# Sex and Age Disparities in the Prevalence of Obesity Among Children and Adolescents in Ghana, 1990–2022: A Cross-Sectional Study

**DOI:** 10.3390/nu18132050

**Published:** 2026-06-23

**Authors:** Richard Gyan Aboagye, Joshua Okyere, Franklin Akwasi Adjei, Blessing Jaka Akombi-Inyang

**Affiliations:** 1 School of Population Health, University of New South Wales, Sydney, NSW 2052, Australia; b.akombi@unsw.edu.au; 2 Department of Allied Health Professions, Sport and Exercise, School of Human and Health Sciences, University of Huddersfield, Queensgate, Huddersfield HD1 3DH, UK; joshuaokyere54@gmail.com; 3 College of Health Sciences, University of Ghana, Accra 23321, Ghana; 4 Division of Kinesiology and Health, College of Health Sciences, University of Wyoming, Laramie, WY 82071, USA; franklinadjei509@gmail.com

**Keywords:** obesity, children, adolescents, inequality, Ghana

## Abstract

**Objective**: This study examined the disparities in the prevalence of obesity among children and adolescents from 1990 to 2022. **Methods:** Crude prevalence estimates were obtained from the World Health Organization’s (WHO) Global Health Observatory, accessible through the WHO Health Equity Assessment Toolkit (WHO HEAT). The study population comprised children and adolescents aged 5 to 19 years. Obesity was defined as a body mass index (BMI) exceeding two standard deviations above the mean, in accordance with the WHO Growth Reference. Descriptive analysis was employed to examine longitudinal trends and disparities in crude obesity prevalence. The dimensions of age (5–9 and 10–19 years) and sex (female and male) were utilised to assess disparities related to obesity. Absolute and relative inequalities were evaluated using difference (D) and ratio (R) summary measures, respectively. **Results:** In 1990, the crude prevalence of obesity was higher among female children and adolescents (1.45%; confidence interval [CI] 0.38–3.41) compared to their male counterparts (1.07%; CI 0.14–3.40). However, by 2022, the prevalence was higher among males (8.20%; CI 5.15–12.01) compared to females (5.78%; CI 3.57–8.48). Regarding age, the prevalence of obesity in 1990 was 2.24% among 5–9-year-olds, compared with 0.59% among 10–19-year-olds. Both age groups saw an increase in crude obesity prevalence over time, and by 2022, the prevalence of obesity was 12.10% among 5–9-year-olds, compared with 4.04% among 10–19-year-olds. In 1990, the difference and ratio estimates were 0.38 and 1.36, respectively, indicating a higher prevalence among females than males. Concurrently, the ratio decreased from 1.36 in 1990 to 0.71 in 2022, further confirming the shift towards a higher prevalence of male obesity in later years. The difference in obesity prevalence (5–9 years minus 10–19 years) stayed positive throughout the study period. In 2022, the age difference in crude obesity prevalence was +8.07 percentage points, and the ratio was 3.00, indicating that the younger group had a prevalence three times that of the older group. **Conclusions:** The prevalence of childhood and adolescent obesity increased significantly from 1990 to 2022, with a shift from females to males and a disproportionate impact on younger children. These trends underscore the necessity for targeted public health interventions that address age- and sex-specific disparities.

## 1. Introduction

Obesity remains a significant public health concern globally, particularly among children and adolescents [1]. In low- and middle-income countries, the rise in obesity has become increasingly evident in recent decades, mirroring trends observed in high-income countries following the COVID-19 pandemic [1]. Several countries in sub-Saharan Africa are experiencing a rising burden of obesity [1,2,3], with contributing factors including male sex, larger-than-average birth size or weight, maternal higher education, and regional residence [2]. In 2022, more than 390 million children and adolescents aged 5–19 years were overweight, including 160 million classified as obese [3]. Additionally, approximately 35 million children under the age of five were overweight worldwide in 2024 [3]. 

Available evidence indicates that approximately 19% of children in Ghana are either obese or overweight [2]. A recent study reported the prevalence of overweight and obesity among 23,663 in-school children and adolescents (aged 5–19) in Ghana as ranging from 0.5% to 47.06% [4]. Recently, numerous studies have documented considerable variation in the reported prevalence of childhood overweight and obesity in Ghana, with estimates of obesity ranging from 0.7% to 47.06%, and overweight from 1.8% to 33.66% [5,6,7]. Such disparities may be attributed to differences in the geographic locations where these studies were conducted, as regional dietary patterns, food availability, and lifestyle behaviours vary significantly across the country [8,9]. Consequently, most indigenous inhabitants consume energy-dense foods to carry out their daily activities, which have been identified as potential factors contributing to the high prevalence of obesity [8,9]. However, current data from Ghana appear fragmented and predominantly drawn from region-specific studies, resulting in varied prevalence estimates and limited understanding of national patterns and trends over time. Unlike high-income countries with ongoing long-term data [6], there is limited data on how obesity among Ghanaian children and adolescents has evolved historically or whether sex differences follow similar trajectories. This paucity of detailed, sex- and age-specific long-term data restricts comprehensive understanding of childhood and adolescent obesity in Ghana. Analysing these temporal trends is vital for understanding the extent and progression of obesity disparities within this population.

Sex disparities in obesity rates are commonly observed worldwide, with females being associated with twice the risk of being obese as males [10]. Ghana is no exception, where girls show a higher prevalence of obesity compared to boys [4]. Several studies have indicated sex-specific differences in prevalence and associated risk factors [2,6,11]. For instance, females are more likely to experience obesity than males, and the prevalence of obesity is approximately 50% higher for females than for males [11]. Biological, behavioural, and psychosocial factors explain these sex disparities. Biologically, women have a higher proportion of total body fat and a different fat distribution pattern, including both subcutaneous and visceral fat deposits, which are associated with an increased risk of metabolic complications [12]. Certain behavioural factors, such as physical inactivity, dietary patterns, and psychosocial stressors, increase obesity risk among women [13]. Psychosocial factors also impact outcomes, with women experiencing higher rates of obesity-related health issues and mortality compared to men [14].

Despite substantial evidence concerning sex differences in obesity, certain gaps persist within the existing literature. One such gap is that numerous studies predominantly rely on cross-sectional data, which limits the ability to establish causality or understand temporal trends [15]. Furthermore, there is a paucity of longitudinal and intervention studies that examine how sex disparities change over time or respond to targeted interventions, thereby limiting the formulation of evidence-based recommendations [16,17]. Addressing these deficiencies requires the application of longitudinal and trend-based analyses to better comprehend the development and progression of sex disparities in obesity. Without such data, it remains uncertain whether differences between boys and girls are stable, increase, or decrease over age and time. Additionally, in Ghana, there is a significant scarcity of research systematically evaluating sex-specific obesity trends over time. Analysing these patterns longitudinally can identify periods when sex differences are most prominent and highlight the groups most affected. 

Age constitutes a critical dimension in obesity research, as the risk, prevalence, and consequences of obesity vary across different developmental stages. Childhood and adolescence represent distinct biological and behavioural phases characterised by variations in growth, hormonal fluctuations, physical activity, and dietary patterns—factors that influence obesity risk [7,11]. Evidence indicates that obesity prevalence tends to increase with age during adolescence; however, the timing and extent of these changes differ across populations [7]. Nonetheless, few studies have systematically examined age-specific obesity patterns or their temporal trends in Ghana. The absence of age-disaggregated evidence hampers understanding of when obesity risk manifests and which developmental stages are most vulnerable. Consequently, this study investigates obesity trends by sex and age among children and adolescents in Ghana from 1990 to 2022, addressing a significant research gap.

By analysing obesity trends by age and sex, this study provides empirical evidence on whether disparities between boys and girls have changed over time, and at which ages these differences are most pronounced. Such trend-based evidence is necessary to clarify the epidemiological patterns of childhood and adolescent obesity. Documenting these sex- and age-specific trajectories contributes to a more precise understanding of obesity dynamics and provides a foundation for future research examining the mechanisms underlying these disparities.

## 2. Methods

### 2.1. Data Source and Design

We conducted a trend analysis using secondary data from the World Health Organization’s (WHO) Global Health Observatory [18,19], which has been integrated into the WHO Health Equity Assessment Toolkit (HEAT) [18,19,20]. The data were originally sourced from population-based surveys, including the WHO STEPwise and other surveillance surveys [19,21], and subsequently collated by the WHO as estimates within the HEAT software (version 6.0). These sources utilised standardised criteria to collect data from the respondents. The WHO subsequently generates estimates of the crude prevalence of obesity and makes them available through the HEAT. For this study, we utilised these estimates to examine age- and sex-related disparities in the crude prevalence of obesity among children and adolescents in Ghana from 1990 to 2022. This approach aligns with the study’s goal of analysing temporal trends and population-level disparities in obesity prevalence. Each year provides an independent population estimate based on separate data sources and models, enabling comparisons over time.

### 2.2. Measures

Prevalence of obesity in children and adolescents was the outcome variable. Obesity was defined as a body mass index (BMI) greater than 2 standard deviations above the mean in children and adolescents, based on the WHO standard reference [22].

Two inequality dimensions were available in the WHO HEAT for segregating the prevalence of obesity among children and adolescents. The two dimensions were sex and age. The categories for sex were ‘male’ and ‘female’, whereas those of age were ‘5–9 years’ and ‘10–19 years’.

We used the difference (D) and ratio (R) summary measures to analyse the crude sex and age disparities in crude obesity prevalence among children and adolescents [23]. D and R were selected to examine inequalities in crude obesity prevalence because they were available in the WHO HEAT and relevant to the literature [23]. D was an absolute summary measure, whereas R was a relative measure of inequality. D measured the difference in obesity prevalence between females and males, as well as between the 5–9-year and 10–19-year groups. Regarding sex, D equalled the estimate in females minus the estimate in males. For age, D was calculated as the estimate in the 5–9-year group minus the estimate in the 10–19-year group. In the absence of inequality, D equals zero, whereas larger absolute values indicate a high level of inequality [23].

On the other hand, R was calculated as the prevalence of obesity in the female group divided by that in the male group, revealing a relative sex disparity in obesity. In terms of age, R was estimated by dividing the prevalence of obesity in the 5–9-year group by the 10–19-year group. R assumes only positive values. In the absence of inequality, R equals one (1). The value of R should be greater than 1 for inequality to exist, with higher values indicating greater inequality [23].

### 2.3. Statistical Analyses

We used the online version of the WHO HEAT 6.0 [18,20] to generate crude sex- and age-specific prevalence of obesity among children and adolescents from 1990 to 2022. We generated the study estimates in two phases. In the first phase, we utilised the disaggregated estimates to present the crude age- and sex-specific obesity prevalence from 1990 to 2022. The second phase involved generating estimates of the absolute and relative summary measures. Two summary measures, one absolute and one relative, were used to examine the extent of inequality in obesity prevalence. The estimates generated from the WHO HEAT online platform were exported to Microsoft Excel for analysis and presentation of results. We used figures to present the results, showing the trends in obesity among males and females, as well as for the 5–9-year and 10–19-year groups. The results of the summary measures were presented using tables.

### 2.4. Ethics Consideration

Ethics approval was not required because the study used secondary data available in the public domain. The WHO aggregated these estimates from the population-based surveys, including the WHO STEPwise and other surveillance surveys, into the WHO HEAT, which was used to generate the estimates for the study.

## 3. Results

### 3.1. Temporal Trends of Obesity Among Children and Adolescents by Sex

**Figure 1:**
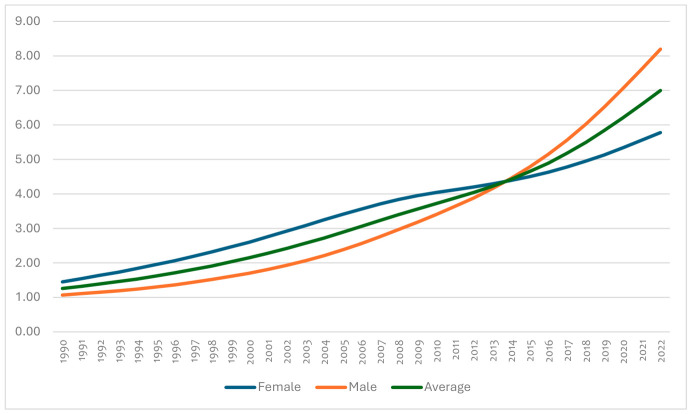
Crude prevalence of obesity by sex.

The overall average prevalence of obesity among children and adolescents in Ghana increased from 1.26% in 1990 to 7.00% in 2022. Between 1990 and 2022, the crude obesity prevalence increased in both sexes. The crude obesity prevalence among females rose from 1.45% (95% confidence interval [CI], 0.38–3.41) in 1990 to 5.78% (95% CI, 3.57–8.48) in 2022. Among male children and adolescents, the crude prevalence increased from 1.07% (95% CI, 0.14–3.40) in 1990 to 8.20% (95% CI, 5.15–12.01). Females continued to show higher crude obesity prevalence than males until 2014 (females [4.39%] vs. males [4.45%]), when the male prevalence estimate exceeded that of females; this trend continued through to 2022 (Figure 1 and Supplementary Table S1).

### 3.2. Temporal Trends in Obesity Among Children and Adolescents by Age

**Figure 2:**
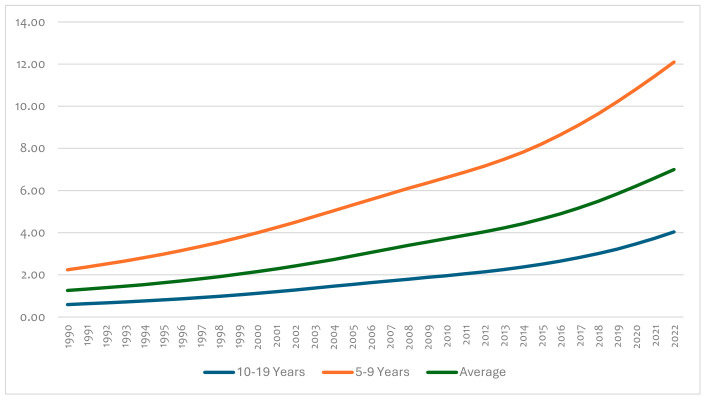
Crude prevalence of obesity by age.

Throughout the study period (1990–2022), children and adolescents aged 5–9 consistently had a higher crude obesity prevalence than the 10–19-year group. In 1990, the prevalence of obesity was 2.24% (CI 0.77–4.94) among 5–9-year-olds, compared with 0.59% (CI 0.18–1.36) among 10–19-year-olds. Both age groups saw an increase in crude obesity prevalence over time, and the gap persisted. The 5–9 year group showed the steepest increase in crude obesity prevalence, reaching double figures in 2019 (5–9 [10.21%] vs. 10–19 [3.22%]). By 2022, obesity was 12.10% (CI 8.95–15.65) in 5–9-year-olds compared to 4.04% (CI 2.75–5.62) in 10–19-year-olds (Figure 2 and Supplementary Table S2).

### 3.3. Sex Inequalities in Obesity Prevalence Among Children and Adolescents in Ghana

In 1990, the difference (female–male) was 0.38 percentage points, and the ratio (female/male) was 1.36, indicating a higher prevalence among females. The difference remained positive until 2013, then became negative (−0.14) and continued to decrease, reaching −2.42 in 2022. Concurrently, the ratio decreased from 1.36 in 1990 to 0.71 in 2022, indicating a higher male crude obesity prevalence in later years (Table 1).

### 3.4. Age Inequalities in Obesity Prevalence Among Children and Adolescents in Ghana

The findings indicate that, despite an overall increase in obesity across both demographic groups, children aged 5–9 consistently bore a disproportionately higher burden of obesity. Age groups 5–9 years and 10–19 years remained positive throughout the study period. In 2022, the age difference in crude obesity prevalence was +8.07 percentage points, with a ratio of 3.00, signifying that the prevalence of obesity in the 5–9-year age group was three times that of the 10–19-year age group (Table 2).

## 4. Discussion

In the last 33 years, the prevalence of obesity among children and adolescents (5–19 years) has increased from 1.26% in 1990 to 7.00% in 2022. The observed increase in childhood and adolescent obesity is consistent with global trends, which have increased by 6% between 2000 and 2022 [24]. While longitudinal trend studies among children and adolescents aged 5–19 are limited in Ghana, our findings align with evidence from Ghana showing an increase in obesity among older adolescents [25,26]. Several factors may account for the increasing trend in obesity among children and adolescents in Ghana. One such factor is the increasing trend of urbanisation in Ghana. The Ghana Statistical Service [27] reports that the country’s urban population increased from 50.9% in 2010 to 56.7% in 2021. Urbanisation often amplifies exposure to obesogenic environments, including greater availability of ultra-processed foods, higher consumption of sugar-sweetened beverages, and reduced opportunities for physical activity [26,28]. For example, Ribeiro et al. [28] found that in urban areas, the availability of fast-food restaurants within walking distance of the child’s residence increased the odds of obesity by 37%. A scoping review of the risk factors for childhood obesity in Ghana cites high levels of television viewing and computer game playing as key drivers of the increasing trend [4].

Our findings revealed a unique trend among Ghanaian children and adolescents. In the initial years, females had a higher prevalence than males. However, by 2022, this trend had reversed: males reported a higher prevalence of obesity than females. While we find no study that directly mirrors this observation, the current higher prevalence of obesity among male children and adolescents is consistent with evidence from India [29] and China [30]. Wang et al. [30] revealed that adolescent boys were twice as likely to be obese as girls. We argue that the observed shift from female to male predominance may reflect changes in the major risk factors of obesity. In the late 90s and early 2000s, video gaming was not reported as a key risk factor of obesity among children and adolescents in Ghana. However, evidence now suggests that playing video/computer games significantly exacerbates the risk of obesity [4]. Evidence from Ghana shows that males spend more time playing video games than females [31], potentially explaining the recent male predominance in obesity prevalence. Beyond gaming, males’ low participation in domestic chores may contribute to incidental physical inactivity and exacerbate their risk of obesity compared to that of females.

Younger children (5–9 years) consistently exhibited substantially higher obesity rates than adolescents (10–19 years). This is inconsistent with an earlier study conducted in Ghana, which found a higher prevalence among adolescents aged 11–16 years (30.6%) than among children aged 5–10 years (13.1%) [32]. Ganle et al. [32]’s study was conducted in an urban area in Ghana, whereas the current study covers the entire country, which has considerable variation in socio-demographic and socio-cultural characteristics. Nevertheless, it aligns with the global picture, which estimates a higher prevalence of childhood obesity among those aged 5–9 years than among those aged 10–19 years [33]. This finding suggests that the early years of childhood are a particularly vulnerable period for the development of obesity in Ghana. Possible factors contributing to this early onset and higher prevalence in younger children include early dietary habits, such as the introduction of processed foods, limited opportunities for active play, and parental influences on lifestyle choices [34].

### 4.1. Implications for Policy and Practice

The rising prevalence of obesity, coupled with the observed shifts in sex and persistent age disparities, necessitates a multifaceted and targeted approach. The Ministry of Health, through the Ghana Health Service, should initiate a national campaign to raise public awareness of the increasing prevalence of obesity among children and adolescents in the country. Public health campaigns must be tailored to address the specific needs of different age groups and sexes. In practice, interventions aimed at younger children might focus on promoting healthy eating habits within families and encouraging active play, while those for adolescents could address sedentary screen time and access to nutritious food options in school settings. The increasing male obesity prevalence highlights the need to move beyond traditional assumptions and develop interventions that resonate with and are effective for boys and young men.

### 4.2. Strengths and Limitations

Our study is the first to analyse a 33-year trend of obesity among children and adolescents in Ghana. It also uniquely examines sex and age disparities in the prevalence of obesity from 1990 to 2022. However, there are inherent limitations that must be acknowledged. This study used only national-level estimates from the WHO Global Health Observatory (WHO HEAT), which precluded the examination of regional prevalence disparities in obesity among children and adolescents. The estimates from the WHO HEAT were unadjusted (crude), which may overestimate or underestimate the true prevalence of obesity; therefore, the findings should be interpreted with caution, as the prevalence estimates may have varied had sociodemographic and socioeconomic factors been controlled for. Additionally, data on risk factors and nutritional components were unavailable for further analysis. Furthermore, single-year age-specific estimates were unavailable in the WHO HEAT. The estimates were available only for the aggregated age groups of 5–9 and 10–19 years, limiting our study’s ability to analyse trends for specific ages. Moreover, the WHO Global Health Observatory crude obesity prevalence estimates used in this study did not include the standard errors required by the WHO HEAT software to calculate confidence intervals for D and R; therefore, inferences drawn from the results should be interpreted in light of this limitation.

## 5. Conclusions

This study provides the first long-term (33-year) nationally representative analysis of childhood and adolescent obesity trends in Ghana, disaggregated by both sex and age group. It documents a reversal in sex disparities, highlighting that boys have now overtaken girls in obesity prevalence. This challenges prevailing assumptions in Ghana’s obesity discourse and underscores the need for interventions targeting both sexes. Moreover, the finding of persistently higher prevalence in 5–9-year-olds calls for early prevention efforts, starting before adolescence, when behavioural and dietary habits are more modifiable.

## Figures and Tables

**Table 1:** Sex inequalities in crude obesity prevalence by summary measures.

Year	Difference (Estimate)	Ratio (Estimate)
2022	−2.42	0.71
2021	−2.06	0.73
2020	−1.72	0.76
2019	−1.39	0.79
2018	−1.08	0.82
2017	−0.78	0.86
2016	−0.52	0.90
2015	−0.28	0.94
2014	−0.06	0.99
2013	0.14	1.03
2012	0.32	1.08
2011	0.49	1.13
2010	0.64	1.19
2009	0.77	1.24
2008	0.87	1.29
2007	0.95	1.34
2006	1.00	1.39
2005	1.03	1.43
2004	1.03	1.47
2003	1.02	1.49
2002	0.99	1.51
2001	0.95	1.52
2000	0.90	1.53
1999	0.85	1.53
1998	0.80	1.53
1997	0.75	1.52
1996	0.70	1.51
1995	0.65	1.50
1994	0.60	1.48
1993	0.54	1.46
1992	0.49	1.43
1991	0.43	1.39
1990	0.38	1.36

**Table 2:** Age inequalities in crude obesity prevalence by summary measures.

Year	Difference (Estimate)	Ratio (Estimate)
2022	8.07	3.00
2021	7.71	3.06
2020	7.34	3.12
2019	6.98	3.17
2018	6.64	3.21
2017	6.30	3.24
2016	5.99	3.26
2015	5.71	3.28
2014	5.45	3.30
2013	5.22	3.32
2012	5.02	3.34
2011	4.83	3.36
2010	4.66	3.38
2009	4.49	3.39
2008	4.31	3.40
2007	4.13	3.41
2006	3.94	3.42
2005	3.76	3.43
2004	3.57	3.45
2003	3.39	3.47
2002	3.21	3.50
2001	3.03	3.52
2000	2.87	3.55
1999	2.71	3.58
1998	2.56	3.60
1997	2.42	3.62
1996	2.29	3.65
1995	2.16	3.67
1994	2.05	3.69
1993	1.94	3.71
1992	1.84	3.74
1991	1.74	3.77
1990	1.65	3.80

## Data Availability

The estimates generated and/or analysed in this study can be accessed at https://www.who.int/data/inequality-monitor/data (accessed on 1 July 2025).

## Supplementary Materials

The following supporting information can be downloaded at: https://www.mdpi.com/article/10.3390/nu18132050/s1, Table S1: Crude prevalence of obesity segregated by sex; Table S2: Crude prevalence of obesity segregated by age.

## Abbreviations

CI	Confidence interval
D	Difference
HEAT	Health Equity Assessment Toolkit
R	Ratio
WHO	World Health Organization

## References

[1] Jebeile H., Kelly A.S., O’Malley G., Baur L.A. (2022). Obesity in children and adolescents: Epidemiology, causes, assessment, and management. Lancet Diabetes Endocrinol..

[2] Akowuah P.K., Kobia-Acquah E. (2020). Childhood obesity and overweight in Ghana: A systematic review and meta-analysis. J. Nutr. Metab..

[3] World Health Organization (2025). Obesity and Overweight.

[4] Amoadu M., Obeng P., Abekah Baah J., Acquah P., Cobbinah G., Aku Ogum M., Owusu Sarfo J., Wilson Ansah E. (2024). Overweight and Obesity Among In-School Children and Adolescents (5–19 Years) in Ghana: A Scoping Review of Prevalence and Risk Factors. J. Obes..

[5] Amoh I., Appiah-Brempong E. (2017). Prevalence and risk factors of obesity among senior high school students in the Adansi North district of Ghana. Int. J. Community Med. Public Health.

[6] Atsu B.K., Guure C., Laar A.K. (2017). Determinants of overweight with concurrent stunting among Ghanaian children. BMC Pediatr..

[7] Annan-Asare J., Asante M., Amoah A.G. (2017). Obesity and its correlates among Junior High School children in the Accra Metropolis. J. Nutr. Health Sci..

[8] Abizari A.R., Ali Z. (2019). Dietary patterns and associated factors of schooling Ghanaian adolescents. J. Health Popul. Nutr..

[9] Frank L.K., Kröger J., Schulze M.B., Bedu-Addo G., Mockenhaupt F.P., Danquah I. (2014). Dietary patterns in urban Ghana and risk of type 2 diabetes. Br. J. Nutr..

[10] Kapoor N., Arora S., Kalra S. (2021). Gender disparities in people living with obesity-an unchartered territory. J. Mid-Life Health.

[11] Ameye H., Swinnen J. (2019). Obesity, income and gender: The changing global relationship. Glob. Food Secur..

[12] Shapira N. (2013). Women’s higher health risks in the obesogenic environment: A gender nutrition approach to metabolic dimorphism with predictive, preventive, and personalised medicine. EPMA J..

[13] Mouchacca J., Abbott G.R., Ball K. (2013). Associations between psychological stress, eating, physical activity, sedentary behaviours and body weight among women: A longitudinal study. BMC Public Health.

[14] Eisenberg K.N., Leiter E., May R.T., Reinfeld T., Zwas D.R. (2020). Psychosocial functioning, BMI, and nutritional behaviors in women at cardiovascular risk. Front. Psychol..

[15] Lombardo M., Feraco A., Armani A., Camajani E., Gorini S., Strollo R., Padua E., Caprio M., Bellia A. (2024). Gender differences in body composition, dietary patterns, and physical activity: Insights from a cross-sectional study. Front. Nutr..

[16] Kantowski T., Schulze zur Wiesch C., Aberle J., Lautenbach A. (2024). Obesity management: Sex-specific considerations. Arch. Gynecol. Obstet..

[17] Pagoto S.L., Schneider K.L., Oleski J.L., Luciani J.M., Bodenlos J.S., Whited M.C. (2012). Male inclusion in randomized controlled trials of lifestyle weight loss interventions. Obesity.

[18] World Health Organization (2024). WHO Health Inequality Monitor Data Repository. https://www.who.int/data/inequality-monitor/data.

[19] World Health Organization (2025). The Global Health Observatory–Obesity Among Children and Adolescents, BMI > +2 Standard Deviations Above the Median, Prevalence (Crude Estimate) (%). https://www.who.int/data/gho/data/indicators/indicator-details/GHO/prevalence-of-obesity-among-children-and-adolescents-bmi-2-standard-deviations-above-the-median-(crude-estimate)-(-).

[20] World Health Organization (2024). Health Equity Assessment Toolkit (HEAT): Software for Exploring and Comparing Health Inequalities in Countries and Territories.

[21] Phelps N.H., Singleton R.K., Zhou B., Heap R.A., Mishra A., Bennett J.E., Paciorek C.J., Lhoste V.P., Carrillo-Larco R.M., Stevens G.A. (2024). Worldwide trends in underweight and obesity from 1990 to 2022: A pooled analysis of 3663 population-representative studies with 222 million children, adolescents, and adults. Lancet.

[22] Onis M.D., Onyango A.W., Borghi E., Siyam A., Nishida C., Siekmann J. (2007). Development of a WHO growth reference for school-aged children and adolescents. Bull. World Health Organ..

[23] Schlotheuber A., Hosseinpoor A.R. (2022). Summary measures of health inequality: A review of existing measures and their application. Int. J. Environ. Res. Public Health.

[24] Muyulema S.L., Carpio-Arias T.V., Verdezoto N., Lara V.E., Manzano A.S., Pulgar H., Veloz M.F. (2025). Worldwide trends in childhood overweight and obesity over the last 20 years. Clin. Nutr. ESPEN.

[25] Okyere J. (2025). Trends and economic inequalities in obesity prevalence in Ghana: A cross-sectional study spanning 2008–2022. J. Health Popul. Nutr..

[26] Tuoyire D.A. (2021). Overweight/obesity among 15-to 24-year-old women in Ghana: 21-year trend, future projections and socio-demographic correlates. J. Biosoc. Sci..

[27] Ghana Statistical Service (GSS) (2021). Ghana 2021 Population and Housing Census General Report: Age and Sex Profile.

[28] Ribeiro A.I., Santos A.C., Vieira V.M., Barros H. (2020). Hotspots of childhood obesity in a large metropolitan area: Does neighbourhood social and built environment play a part?. Int. J. Epidemiol..

[29] Singh S., Awasthi S., Kapoor V., Mishra P. (2023). Childhood obesity in India: A two-decade meta-analysis of prevalence and socioeconomic correlates. Clin. Epidemiol. Glob. Health.

[30] Wang V.H., Min J., Xue H., Du S., Xu F., Wang H., Wang Y. (2018). What factors may contribute to sex differences in childhood obesity prevalence in China?. Public Health Nutr..

[31] Miezah D., Batchelor J., Megreya A.M., Richard Y., Moustafa A.A. (2020). Video/computer game addiction among university students in Ghana: Prevalence, correlates and effects of some demographic factors. Psychiatry Clin. Psychopharmacol..

[32] Ganle J.K., Boakye P.P., Baatiema L. (2019). Childhood obesity in urban Ghana: Evidence from a cross-sectional survey of in-school children aged 5–16 years. BMC Public Health.

[33] Lobstein T., Brinsden H. (2019). Atlas of Childhood Obesity.

[34] Aboagye R.G., Kugbey N., Ahinkorah B.O., Seidu A.A., Cadri A., Bosoka S.A., Akonor P.Y., Takase M. (2022). Nutritional status of school children in the South Tongu District, Ghana. PLoS ONE.

