# Supplementary material for: Sex and Age Disparities in the Prevalence of Obesity Among Children and Adolescents in Ghana, 1990–2022: A Cross-Sectional Study
Source: Nutrients. 2026 Jun 23;18(13):2050. doi: 10.3390/nu18132050 (PMC13363566; doi:10.3390/nu18132050)
Supplement: Supplementary file 1 [file nutrients-18-02050-s001.zip › nutrients-4227718-supplementary.pdf]

SUPPLEMENTARY FILE

Sex and Age Disparities in the Prevalence of Obesity Among Children and Adolescents in Ghana, 1990–2022: A Cross-Sectional Study

Table S1: Crude prevalence of obesity segregated by sex

Year	Female			Male			Average
	Estimate	CI LB	CI UB	Estimate	CI LB	CI UB	Estimate
2022	5.78	3.57	8.48	8.20	5.15	12.01	7.00
2021	5.56	3.65	7.89	7.62	5.08	10.66	6.60
2020	5.34	3.66	7.34	7.06	4.94	9.50	6.21
2019	5.13	3.64	6.89	6.52	4.75	8.46	5.84
2018	4.95	3.60	6.51	6.02	4.51	7.71	5.49
2017	4.78	3.53	6.20	5.56	4.20	7.11	5.18
2016	4.63	3.45	5.97	5.15	3.81	6.70	4.89
2015	4.50	3.35	5.81	4.78	3.42	6.42	4.65
2014	4.39	3.24	5.68	4.45	3.01	6.19	4.42
2013	4.29	3.12	5.61	4.15	2.63	5.99	4.22
2012	4.20	3.03	5.54	3.88	2.29	5.89	4.04
2011	4.12	2.93	5.52	3.64	1.98	5.78	3.88
2010	4.04	2.85	5.48	3.40	1.71	5.71	3.72
2009	3.95	2.74	5.42	3.18	1.49	5.59	3.56
2008	3.84	2.63	5.31	2.97	1.28	5.47	3.40
2007	3.71	2.49	5.22	2.76	1.10	5.29	3.23
2006	3.56	2.34	5.10	2.56	0.95	5.13	3.06
2005	3.41	2.20	4.95	2.38	0.82	4.91	2.89
2004	3.25	2.04	4.80	2.21	0.71	4.74	2.72
2003	3.08	1.89	4.65	2.06	0.62	4.59	2.57
2002	2.92	1.74	4.48	1.93	0.54	4.48	2.42
2001	2.76	1.59	4.37	1.81	0.47	4.31	2.28
2000	2.60	1.44	4.20	1.70	0.42	4.17	2.15
1999	2.46	1.29	4.07	1.61	0.37	4.02	2.03
1998	2.32	1.15	3.97	1.52	0.33	3.92	1.91
1997	2.19	1.01	3.90	1.44	0.31	3.84	1.81
1996	2.06	0.90	3.80	1.36	0.28	3.75	1.71
1995	1.95	0.79	3.75	1.30	0.24	3.66	1.62
1994	1.84	0.68	3.65	1.24	0.22	3.62	1.53
1993	1.73	0.60	3.61	1.19	0.20	3.51	1.46
1992	1.64	0.53	3.57	1.15	0.18	3.47	1.39
1991	1.54	0.45	3.48	1.11	0.16	3.45	1.32
1990	1.45	0.38	3.41	1.07	0.14	3.40	1.26

CI: Confidence interval, LB: Lower boundary, UB: Upper boundary

Table S2: Crude prevalence of obesity segregated by age

Year	10-19 Years			5-9 Years			Average
	Estimate	CI LB	CI UB	Estimate	CI LB	CI UB	
2022	4.04	2.75	5.62	12.10	8.95	15.65	7.00
2021	3.74	2.68	5.01	11.44	8.77	14.43	6.60
2020	3.47	2.60	4.48	10.81	8.51	13.36	6.21
2019	3.22	2.49	4.06	10.21	8.19	12.36	5.84
2018	3.01	2.37	3.73	9.64	7.83	11.60	5.49
2017	2.82	2.24	3.48	9.12	7.43	10.98	5.18
2016	2.65	2.09	3.27	8.64	6.98	10.45	4.89
2015	2.50	1.94	3.13	8.20	6.53	10.06	4.65
2014	2.37	1.82	3.00	7.81	6.09	9.73	4.42
2013	2.25	1.69	2.90	7.47	5.66	9.49	4.22
2012	2.14	1.58	2.82	7.16	5.29	9.29	4.04
2011	2.05	1.47	2.76	6.88	4.94	9.14	3.88
2010	1.96	1.38	2.69	6.62	4.65	9.00	3.72
2009	1.88	1.30	2.61	6.36	4.35	8.84	3.56
2008	1.79	1.21	2.54	6.11	4.07	8.66	3.40
2007	1.71	1.13	2.46	5.84	3.80	8.49	3.23
2006	1.63	1.05	2.40	5.57	3.54	8.28	3.06
2005	1.54	0.97	2.30	5.30	3.27	8.04	2.89
2004	1.46	0.89	2.20	5.03	3.02	7.80	2.72
2003	1.37	0.82	2.12	4.76	2.79	7.53	2.57
2002	1.28	0.74	2.03	4.49	2.56	7.32	2.42
2001	1.20	0.67	1.95	4.24	2.36	7.02	2.28
2000	1.12	0.61	1.87	3.99	2.17	6.74	2.15
1999	1.05	0.55	1.80	3.76	1.99	6.52	2.03
1998	0.98	0.49	1.74	3.54	1.82	6.28	1.91
1997	0.92	0.44	1.69	3.34	1.67	6.13	1.81
1996	0.86	0.39	1.62	3.15	1.53	5.92	1.71
1995	0.81	0.35	1.57	2.97	1.38	5.71	1.62
1994	0.76	0.31	1.52	2.81	1.24	5.45	1.53
1993	0.71	0.27	1.46	2.65	1.12	5.32	1.46
1992	0.67	0.24	1.42	2.51	0.98	5.20	1.39
1991	0.63	0.21	1.37	2.37	0.87	5.04	1.32
1990	0.59	0.18	1.36	2.24	0.77	4.94	1.26

CI: Confidence interval, LB: Lower boundary, UB: Upper boundary
